# Preliminary Observations on Skin Disinfection and Wound Infection1Read before the Thirty-eighth Annual Meeting of the American Veterinary Medical Association.

**Published:** 1901-10

**Authors:** Veranus A. Moore

**Affiliations:** Ithaca, N. Y.


### PRELIMINARY OBSERVATIONS ON SKIN DISINFECTION AND WOUND INFECTION.[1]

By Veranus A. Moore,
ITHACA, N. Y.

It has been my privilege during the last few years to study somewhat closely and to have certain investigations made concerning the bacteria of the skin, the efficiency of certain disinfectants, and the bacterial contents of various wound infections, more espe-

[1] Read before the Thirty-eighth Annual Meeting of the American Veterinary Medical Association.

cially in the horse. While our work in these lines is but fairly begun, the results already obtained seem to warrant a discussion of the preliminary findings in so far as they bear upon antiseptic and aseptic methods. In carrying on the investigations I wish to acknowledge the aid received from Drs. R. J. Stanclift, C. W. Gay, and E. C. Shaw, who, as students in advanced bacteriology, have rendered much valuable assistance. I desire to express my indebtedness to Dr. W. L. Williams for his hearty co-operation and for furnishing material from his surgical clinic.

In the first application of bacteriology to comparative medicine, it was natural that investigations should have been made respecting the etiology of the more serious epizoötic diseases. However, the significance of bacteria in wound infections claimed early attention in human surgery, and it is along the line of asepsis that the teachings of bacteriology are at the present time accomplishing, perhaps, the greatest amount of good. In veterinary surgery, however, antisepsis and asepsis seem to have received less attention than they rightfully demand. The fact is recognized that the domesticated animals are, as a class, less susceptible to invading microörganisms than man, and consequently the cause for the quite prevalent assertion among practitioners that in the lower animals there is little or no need for antiseptic precautions. Nevertheless, the not uncommon infections with serious loss warrant an inquiry into the entire subject of skin disinfection as applied to these animals.

My attention was first called to the desirability of a better knowledge of the bacteria of the skin and the efficiency of some commonly used disinfectants in studying the cause of certain lesions following surgical operations where the prescribed disinfecting methods were carefully followed. An inquiry into the source of the bacterial findings in other chronic lesions more distant from the seat of skin laceration or injury, but which seemed to be traceable in them, suggested still further the importance of this knowledge. The more interesting of these chronic disorders of a local nature are those commonly known as scirrhus cord or botryomycosis, emphalophlebitis, infectious cellulitis, the commonly called "foot-rot" of sheep and other though similar lesions.[1] In taking all of these operative and traumatic disorders into the list of wound

[1] I described, American Veterinary Review, June, 1898, an infectious cellulitis in cattle due to a streptococcus. A disease reported as "foot-rot" in sheep has been investigated and found to be a cellulitis resulting in suppuration, in some cases above and in others under the hoof. In the latter instance the hoof changed as a secondary result —American Veterinary Review, February, 1900.

infections, it is necessary to include as wounds all lacerations, incisions, and punctures, whether of sufficient size to be detected or not. In fact, it does not always seem to be necessary to have even a puncture, as blows or intense straining of the integument may be sufficient to liberate into the living tissues bacteria deeply seated in the ducts of glands and possibly the hair-follicles of the skin. The problem before us resolves itself into three quite distinct parts, namely:

1. The determination of the bacterial flora of the skin.
2. The finding of the degree of efficiency of different disinfectants when applied to the integument.
3. The finding of the species of bacteria which produce the more chronic disorders here included in wound infections.

In starting with the existing methods of preparing the field for operation, we find practically the same procedure recommended in veterinary as in human surgery. It is desirable, therefore, to compare the structure of and the bacteria of the skin of man and of the horse in order to determine whether or not the method prescribed for the first is equally applicable for the second. If we follow the results of the everyday operations there seems to be more frequently in veterinary than in human surgery either acute wound infection or chronic and possibly metastatic lesions of a more or less serious nature. The question whether this is due to the operator, or, bacteriologically speaking, ill-considered methods, is waiting for an answer. It is true that veterinary surgeons who follow the prescribed course may very largely prevent these otherwise common sequences, but it is for the prevention of the occasional failure that the best methods are striving.

In comparing the structure of the skin we will, at this time, mention but two features which suggest greater difficulties in preparing the operative field in the horse than in man, namely, (1) the presence of a thicker layer of dead epithelium, and (2) longer and deeper-seated glands and hair-follicles.

Investigations concerning the bacteria of the human skin have been carefully made by a number of workers, among whom may be mentioned Bizzozero (*Virchow's Archiv*, Bd. xcviii.); Mittman (*Ibid.*, Bd. cxiii.); Unna (*Monatshefte für praktische Dermatologie*, 1889, 1890, 1891); Bordoni-Uffreduzzi (*Fortschritte der Medicin*, 1886, S. 151); Fürbringer (*Disinfektion de Hande O. Arzter.*, Wiesbaden, 1888); Preindlsberger (*Zur Kenntniss der Bacterien des Unternagelraumel, u. s. w.*, Wien, 1891); Robb and Ghriskey (*Johns Hopkins Hospital Bulletin*, April, 1892), and Welch (*Trans-*

*actions of the Congress of American Physicians and Surgeons*, 1891, vol. ii.). The researches of Robb and Ghriskey and Welch have shown that, in addition to the varying bacterial flora of the epidermis, there is in the deeper layers within the ducts of glands, and possibly along the hair shafts, a micrococcus belonging to the staphylococcus group, which seems to be a quite constant inhabitant of the integument. It frequently appeared in surgical wounds, and while it seemed to be possessed with little pathogenesis it was often the apparent etiological factor in stitch abscesses. A very extensive series of examinations of the skin is being made by Dr. W. G. Macdonald, of Albany. His preliminary reference (*Transactions of the Medical Society of the State of New York*, 1900, p. 46) to this work shows that it is not always easy to obtain, in the human subject, a perfectly sterile field of operation.

In the horse we find very similar conditions. The bacterial flora of the untreated skin is very rich in both numbers and species. Like that of the human subject, it is exceedingly variable in different individuals and on different surface areas of the same individual. Technically, however, the bacteria resting simply on the surface of the body, where they have been brought and lodged by various agencies, cannot be considered as belonging to the bacterial flora of the skin. This term should include only the bacteria that are the inhabitants of the deeper layers of the epidermis, the ducts of glands, hair shafts, and follicles. In the horse this flora, which is indefinite and exceedingly variable, consists of a number of generæ of bacteria, but certain micrococci and streptococci seem to predominate, although bacilli are often present. Among the numerous bacteria which have been found in the skin our attention thus far has been given almost entirely to three species which are of interest from the wound infection stand-point. From the continuous exposure of the skin of animals it is not unreasonable at least to presume that many pyogenic and possibly other parasitic bacteria find their way to the deeper crevices of the integument.[1]

The three species of special interest are:

1. A streptococcus which I have been unable to differentiate from a streptococcus found as the apparent cause of septic peritonitis following castration, and in septic pneumonia following other operations.

2. A micrococcus which grows in clumps and forms almost milk-

[1] B. subtilis is very frequently found in the cultures made from the supposed disinfected skins. Its rapid growth, overshadowing other species, renders it exceedingly troublesome in isolating the bacteria of the skin.

white colonies on agar plates. This organism has frequently been isolated from cases of fistulous withers. It was not pathogenic for the experimental animals. It is very similar to the one isolated from the human skin by Robb and Ghriskey.

3. Micrococcus pyogenes aureus. This differs very little from the one described from man. It has been found in closed abscesses (botryomycosis?) apparently as the exciting cause.

It was suggested from our earlier investigations that these three species of skin bacteria were the essential ones, against which skin disinfectants should be more especially directed.[1] Their almost constant appearance in a considerable variety of lesions of probable wound-infection origin gave strength to such a conclusion. Accordingly our inquiries were continued in a series of examinations made with different disinfectants to find if possible the most efficient agents for this purpose. Here, again, our results are but partial, as a number of substances reported as reliable skin disinfectants have as yet not been tried.

The method followed was to make bacteriological examinations of the skin of horses either those to be operated upon or to be used for dissection. In all ten cases may be included, in each of which nine disinfecting solutions were employed on as many different areas, with one untreated area as a check. In preparing the field the hair was clipped, the skin scrubbed with soap and water, closely shaven and washed again with sterile water, after which the disinfecting solution was applied and allowed to act for from eight to ten minutes. Pieces of the treated skin were then removed with sterile instruments and placed in tubes of bouillon and liquid agar, from which plate-cultures were made. The pieces of skin used were about one centimetre square.

A study of the cultures showed the interesting fact that in 80 per cent. of those made from the fields treated with 5 per cent. carbolic acid and with the alcoholic solutions of corrosive sublimate did not contain micrococci and streptococci. The bacilli were not appreciably affected. With the other disinfectants used there was no apparent destruction of the micrococci and streptococci. It is of interest to add that of the seven species isolated from the plates and studied in pure culture two bacilli produced suppurating lesions when injected subcutaneously in the horse. The list of disinfectants, together with the results of the examinations, are given in the appended table:

[1] Specific pathogenic bacteria, such as the bacillus of tetanus, are not included in this discussion, although they are to be taken into account in the final and best methods.

*Table showing the results of disinfectants as indicated by the number of colonies which developed on agar plates made from pieces of skin from areas treated with the different disinfectants.*

| Disinfectants. | Number of cases that gave sterile plates. | Number of cases with few (5 to 30) colonies. | Number of cases where colonies were numerous or very many. | Total. |
|---|---|---|---|---|
| 5 per ct. carbolic acid . . . | 2 | 3 | 5 | 10 |
| 3 " permanganate of potassium | 0 | 1 | 9 | 10 |
| 3 " lysol . . . . | 0 | 1 | 9 | 10 |
| 33 " sanitas . . . . | 0 | 4 | 6 | 10 |
| 2½ " chloronaptholeum . . | 0 | 2 | 8 | 10 |
| 1–1000 bichloride of mercury aq. sol. | 0 | 1 | 9 | 10 |
| 1–500 mercury aq. sol. . . . | 0 | 1 | 9 | 10 |
| 1–1000 " alc. " . . . | 1 | 1 | 8 | 10 |
| 1–500 " " " . . . | 2 | 0 | 8 | 10 |
| Check . . . . . . | 0 | 0 | 10 | 10 |

Without entering into wearisome details, the results of our work on the disinfection of the skin of the horse indicate that the 5 per cent. solution of carbolic acid and the alcoholic solutions of corrosive sublimate are, of the disinfectants tested, those which have a sufficiently penetrating power to destroy, in most cases, the pyogenic micrococci and streptococci in the deeper layers of the integument. Several solutions used more or less commonly as skin disinfectors do not seem, in the strengths employed, to be of value in this connection. It is very likely that it will be difficult if not impossible to find substances that will in all cases sterilize the field for operation.

It is instructive to note the close resemblance if not identity existing between the bacteria of the deeper layers of the skin and those found in the lesions in such affections as septic peritonitis, scirrhous cord, or botryomycosis, closed subcutaneous abscesses, infectious cellulitis, and many other presumably non-specific infections. The bacterial findings in fistulous withers and poll-evil (Moore, *American Veterinary Review*, January, February, and March, 1900 ; Gay, *Ibid.*, March, 1901) show a similar relationship to the pyogenic bacteria of the integument. The hypothesis is suggested by present findings that possibly these troublesome lesions are the result of the setting free into the living tissues, possibly from injuries of many kinds, certain of the bacteria deeply seated in the integument. However, the results of many observations will be necessary before positive conclusions are warranted.

With the other lesions mentioned the possible relation to the bacteria of the skin is more clearly indicated. The infection from the hands of the operator, or from unsterilized instruments is, of course, a very large factor in wound infection in careless or unscientific work, but in modern surgery such accidents should at the most be exceedingly rare. In a few cases which I have examined the infective lesions have followed operations where the usual method of disinfecting the skin was observed, the hands of the operator cleansed and washed in bichloride, and the instruments boiled just prior to the operation. While this does not exclude external infection, it suggests either errors in methods or the presence of the invading microörganisms in the skin of the patient.

The selective power on the part of the horse, or, better, perhaps, that only a certain few species of bacteria which may gain entrance to the fresh living tissues can survive, seems to be well established. This power, if you please so to put it, is well illustrated in case of navel-ill, where the infection unquestionably comes from without and where many species of bacteria are brought in competition. I will describe briefly one of the cases which I have examined in exemplifying the point in question.

A colt, about three weeks old. It was in good condition and seemed to be perfectly well, excepting for the diseased joints. Killed for examination. The umbilical vein, from the umbilicus to the liver, was distended with blood, pus cells, and bacteria. All of the internal organs appeared to be normal. In both knee-joints and in one hock-joint there was extensive suppuration. A bacteriological examination showed the umbilical vein to contain many species of bacteria, among which may be mentioned B. coli communis, micrococcus pyogenes aureus, and a streptococcus.

One of the several tubes of media inoculated from the liver developed the streptococcus, the others remained clear. All media inoculated from the heart-blood, spleen, and kidneys remained sterile. All of the tubes of culture media inoculated with the pus from the joints gave pure cultures of the streptococcus.

The cases of navel-ill which I have seen in lambs were due to an infection with a bacillus belonging to the colon group, and the lesions were diffused subcutaneous and intermuscular suppurative cellulitis.

The lesions known as botryomycosis are, from the etiological stand-point, the most interesting of those here included in wound infections. The cases which form the basis for these remarks were diagnosed clinically by Dr. W. L. Williams, who kindly sent them to me from his clinic for a bacteriological examination. They include the forms known as scirrhous cord and closed abscesses from different parts of the body. There is a difference of opinion concerning the etiology of these lesions. Bollinger, Rivolta, Rabe, and Johne have found in them a peculiar species of microörganism which has been given a variety of names, but generally designated in more recent publications as micrococcus ascoformans. These authors look upon botryomycosis, in consequence of this single causative agent, as a specific disease. A number of works on comparative surgery and pathology treat it as such. On the other hand, Kitt, Hell, de Jong, and Gay have failed to find this organism, but in its stead they isolated micrococcus pyogenes aureus. Whether other bacteria or fungi are involved in these lesions does not seem to be clear.

In my study of this disease I sought diligently for the supposed specific micrococcus ascoformans in cover-glass preparations in many and varied cultures, and in sections of the thickened cord and walls of the closed abscesses, but invariably with negative results. During the last two years I have thus examined six cases reported by the surgeon in charge to be characteristic botryomycosis. Four of these were in the spermatic cord, and two were closed abscesses located elsewhere on the body. From three of these—the two abscesses and one cord—I obtained pure cultures of micrococcus pyogenes aureus. All of the media inoculated from the other three cases remained sterile. These were all of long standing.

The micrococcus obtained from one of the abscesses was very virulent both for rabbits and horses. In the inoculated horses extensive suppurative lesions developed in four days. The rabbits died of septicæmia within thirty-six hours after inoculation.

In another case of infection following castration pure cultures of a streptococcus were obtained from the inflamed cord. This organism, however, cannot be considered as a causative factor in scirrhous cord, as the lesion was too acute and recent to indicate what the final outcome might have been.

In one of the scirrhous cords of long standing, and from which the bacteriological search gave negative results, the firm fibrous tissue of the much thickened part contained numerous pockets filled with a soft, spongy tissue, in which were embedded minute masses of growth resembling the ray fungus of actinomycosis. Sections of this tissue were sent to several pathologists for opinions, and without exception they all pronounced the masses of a fungous nature and probably that of actinomycosis. This is suggestive in the light of the good results obtained in treating this disease with iodide of potash, as reported and advocated by Thomassen and others.

Finally, the conclusion seems to be warranted, at least for a working hypothesis, that botryomycosis is not a specific diseased condition, but that it is the result of infection with any one of several microörganisms, among which Micrococcus pyogenes aureus seems to be the most common. With these somewhat general and simple statements, which are here offered for discussion, the subject must rest until the results of additional and more extended investigations may be recorded.

